# Pregnancy Management in a Patient With Congenital Heart Disease and Severe Right Ventricle to Pulmonary Artery Conduit Stenosis

**DOI:** 10.31486/toj.19.0037

**Published:** 2020

**Authors:** Sajan S. Gill, Kelly K. Shum, Sangeeta B. Shah

**Affiliations:** ^1^ The University of Queensland Faculty of Medicine, Ochsner Clinical School, New Orleans, LA; ^2^ Department of Cardiovascular Disease, John Ochsner Heart and Vascular Institute, Ochsner Clinic Foundation, New Orleans, LA

**Keywords:** *Heart defects–congenital*, *pregnancy*, *pulmonic insufficiency*, *pulmonic stenosis*

## Abstract

**Background:** Pregnancy causes multiple hemodynamic changes that place significant stress on the cardiovascular system. With advancements in medical care, individuals with complex congenital heart disease are living into their childbearing years. Much remains to be understood about the effects and management of pregnancy in individuals with complex congenital heart disease.

**Case Report:** We describe the management and delivery of a 29-year-old pregnant female with repaired tetralogy of Fallot or ventricular septal defect with pulmonary atresia. The patient presented at 21 weeks’ gestation with New York Heart Association class II symptoms and pulmonary conduit stenosis, with a mean gradient of 52 mmHg. At 36.5 weeks’ gestation, she developed severe pulmonary conduit stenosis with a mean gradient of >75 mmHg. The patient was admitted at 37 weeks’ gestation for planned delivery. After a successful cesarean section and bilateral tubal ligation, the patient had an uncomplicated postoperative course. She was scheduled for follow-up for severe conduit stenosis at 6 weeks postpartum to discuss management options.

**Conclusion:** Management of a pregnant patient with adult congenital heart disease should involve risk stratification for complications (commonly congestive heart failure exacerbation and arrhythmias) using tools such as the modified World Health Organization pregnancy risk classification. Based on the risk category, decisions must be made about frequency of follow-up, anesthesia, and mode of delivery. Patients in moderate to high-risk stratification should be managed by a multidisciplinary team at a specialty center, and all patients should undergo an anesthesia consultation prior to delivery. The decision for vaginal or cesarean delivery should be made on a case-by-case basis with consideration given to patient preference. Patients with asymptomatic moderate to severe pulmonic stenosis can be managed conservatively with appropriate follow-up and cardiac imaging, allowing intervention to be completed after delivery.

## INTRODUCTION

Approximately 500,000 women with adult congenital heart disease (ACHD) are estimated to have reached reproductive age in the United States.^1^ As these patients reach childbearing age, a new challenge for the medical field has emerged. Pregnancy results in many hemodynamic changes that may be deleterious to patients with certain congenital abnormalities. Plasma volume increases 40% in the first trimester, coupled with an increase in stroke volume and heart rate, which culminates in a 20% to 50% increase in cardiac output by 24 weeks of gestation^2,3^ and up to 1 mm of aortic dilation.^4^ The placenta, through nitric oxide/prostaglandin production, creates an approximate 35% decrease in systemic vascular resistance and an approximate 10 mmHg decrease in systolic and diastolic pressure.^2,3^

In addition to the physiologic stress on the cardiovascular system throughout pregnancy, delivery of the fetus can be even more challenging for a patient with a tenuous hemodynamic state. With each uterine contraction during vaginal delivery, 300 to 500 cc of blood from the uteroplacental circulation is sent to the systemic circulation.^5^ Immediately following delivery, regardless of the amount of blood loss (an average of 440 mL for cesarean section and 250 mL for vaginal delivery^6^), cardiac output transiently increases 60% to 80% during the first hour.^7^

When patients with ACHD become pregnant, the physiologic stress of pregnancy on a heart with congenital abnormalities may potentially pose a threat to both the mother and thus secondarily to the unborn child. In this case, we report the successful management of a patient with repaired tetralogy of Fallot and subsequent severe pulmonary conduit stenosis during pregnancy and delivery.

## CASE REPORT

A 29-year-old female in her second pregnancy, with a history of tetralogy of Fallot or ventricular septal defect with pulmonary atresia, had undergone 4 surgeries during her childhood, including a modified Blalock-Taussig shunt, patch repair of ventricular septal defect, and a right ventricle (RV) to pulmonary artery (PA) conduit. At 20 weeks’ gestation during her first pregnancy, she developed severe symptomatic stenosis of the RV to PA conduit with a Doppler gradient of 98 mmHg, thus requiring a 22-mm balloon dilatation and Genesis XD stent as a temporizing measure until definitive repair could be undertaken postpartum. However, she was lost to follow-up after delivery of her first child and did not undergo postpartum repair of her RV to PA conduit stenosis.

The patient presented to our clinic during her second pregnancy at 21 weeks of gestation. Thus far, her pregnancy had been uncomplicated and prenatal testing to date had found no abnormalities in fetal development. On presentation, she admitted to mild dyspnea on exertion at a distance of 20 yards. On examination, the patient was tachycardic and normotensive with normal oxygenation. She was euvolemic with hemoglobin of 13.5 g/dL and creatinine of 0.7 mg/dL. Echocardiogram showed normal left ventricular ejection fraction (LVEF) of 60% to 65%, with mild right atrial (RA) dilatation, evidence of RV hypertrophy, and an estimated RV pressure of 82 mmHg. The PA conduit was severely stenosed, with a mean gradient of 52 mmHg and unrestricted pulmonic valve insufficiency. Our assessment determined her modified World Health Organization (WHO) pregnancy risk classification to be II-III (moderately increased risk of maternal mortality and moderate morbidity).

She was seen again in clinic at 34 weeks’ gestation. Her symptoms and repeat echocardiogram were unchanged from the previous visit. However, at 36.5 weeks’ gestation, 2-dimensional echocardiogram done in preparation for delivery identified severe conduit stenosis with a mean gradient of >75 mmHg, peak gradient of 98 mmHg with a peak velocity of 5 m/s, and unrestricted pulmonic valve insufficiency. The patient's RV pressures at the time also exceeded 71 mmHg. Despite the severe restenosis and unrestricted insufficiency of her PA conduit, her symptoms remained New York Heart Association (NYHA) class II. The plan was to admit the patient prior to cesarean section for assessment of heart failure and bilateral tubal ligation at 37 weeks’ gestation.

As planned, she was admitted at 37 weeks for delivery and was cared for by a multidisciplinary team involving obstetrics, ACHD specialists, heart failure specialists, obstetric anesthesia, maternal fetal medicine, and the neonatal intensive care unit.

The patient underwent a successful low transverse cesarean section and bilateral tubal ligation under epidural block with an estimated blood loss of 1,100 mL. Postdelivery echocardiogram showed normal LVEF of 55% to 60%, RA and RV dilatation with RV hypertrophy, and no change in RV pressure or valve gradient. The RV to PA conduit stenosis had decreased to a peak velocity of <4.8 m/s and a mean gradient of <56 mmHg with unrestricted insufficiency.

The patient had an uncomplicated postoperative course, only requiring some gentle hydration, and was discharged 3 days postpartum. She was scheduled for follow-up for severe conduit stenosis at 6 weeks postpartum to discuss management options.

## DISCUSSION

The initial assessment of a patient with ACHD should occur prior to pregnancy, and the physician should determine the risk of proceeding. Multiple models have been developed for this purpose, including the CARPREG (Cardiac Disease in Pregnancy) risk index,^8^ the CARPREG II,^9^ the ZAHARA (Zwangerschap bij Aangeboren Hartaf-wijkingen) score,^10^ and the modified WHO pregnancy risk classification.^3,11^ These scores take into account a patient's cardiac history (history of arrhythmia, stroke, congestive heart failure), NYHA heart failure classification, and current cardiac state (valvular function, severity of pulmonary hypertension, ejection fraction) to provide a cardiac event risk assessment for the pregnancy.^12,13^ The 2017 American Heart Association guidelines recommend the use of the modified WHO pregnancy risk classification.^3^ High-risk groups—patients with Marfan syndrome with aortic root dilation >45 mm, bicuspid aortic valve with aortic dilatation >50 mm, pulmonary hypertension, severe coarctation, severe mitral stenosis or aortic stenosis, and LVEF <30%—are designated as WHO pregnancy risk classification IV and have an extremely high risk of mortality or severe morbidity; as a result, pregnancy is not recommended for these patients.^3^

We determined that our patient was WHO pregnancy risk classification II-III—moderately increased risk of maternal mortality and moderate morbidity—because she had a repaired tetralogy of Fallot with severe pulmonary conduit stenosis.^11^ The European Society of Cardiology 2018 recommendations for this classification are cardiac and obstetric specialist monitoring through pregnancy, delivery, and puerperium.^11^

Many risks are involved as a patient with ACHD progresses through pregnancy. These risks vary by the congenital anomaly, but in general, include congestive heart failure exacerbation, arrhythmias, aortic root dilation leading to rupture, and cardiac death. A 2017 retrospective study of patients with ACHD found that all patients, regardless of ACHD complexity, had higher odds ratios for congestive heart failure development compared to controls: 9.7 and 56.6 for patients with noncomplex and complex congenital heart disease, respectively.^14^ Arrhythmias tend to be the second most common cardiac complication in these patients, with an overall incidence of 4.5%; supraventricular tachycardia was the most common arrhythmia observed, followed by ventricular tachyarrhythmia.^15^ Cardiac death is much rarer, only having been observed as a complication in 1% of pregnancies of mothers with heart disease, congenital and acquired.^8^ The pregnancies of patients with repaired pulmonary atresia and ventricular septal defect resulted in a miscarriage 35% of the time, and 20% of patients developed heart failure.^15^ Additionally, patients with repaired tetralogy of Fallot had an increased rate of cardiac arrhythmia (6.4%) and heart failure (2.4%).^15^

As a part of the initial pregnancy evaluation, a delivery plan should be developed that involves discussions about the necessity for transfer to a specialty center, mode of delivery, and intrapartum anesthesia. Patients with ACHD who are categorized with moderate to high maternal/fetal risk based on the modified WHO pregnancy risk classification should have an anesthesia consultation. Certain indications require a cesarean delivery; in these cases, understanding the effects of epidural anesthesia on patients with ACHD is important. Care must be taken with the use of epidural anesthesia, as it has been found to cause hypotension in >30% of cases secondary to decreases in sympathetic tone.^16^

The mode of delivery also needs to be considered. The European Society of Cardiology 2018 guidelines recommend that patients with pulmonic stenosis undergo vaginal delivery.^11^ Our patient requested tubal ligation at delivery, and thus cesarean section was chosen as the best option for performing both delivery of the fetus and sterilization. The cesarean section also allowed for a controlled delivery setting, as excessive blood loss would lead to reduction in RV preload, which would be detrimental to our patient with RV to PA conduit stenosis. To minimize the risk of deterioration as gestation progressed and ensure availability of the specialist team, delivery was planned at 37 weeks.

The challenge of this specific case was the presence of severe pulmonary conduit stenosis with a mean gradient >75 mmHg in addition to RV hypertrophy and elevated RV pressures. Research on RV to PA conduit stenosis and/or insufficiency during pregnancy is limited; however, some prognostic and management information can be inferred from patients with pulmonic valve disease. Mild and moderate pulmonic stenosis tend to be well tolerated, but if severe pulmonic stenosis is present, follow-up appointments with echocardiography are recommended every 2 months to follow RV function.^17^ When severe pulmonic stenosis becomes symptomatic, similar to when our patient was 20 years old with her first pregnancy, percutaneous pulmonic valve valvuloplasty is recommended.^11^

Once the decision to perform a cesarean section was made, our multidisciplinary team needed to decide on the optimal form of anesthesia: general or regional. Overall anesthetic considerations include control of hypervolemia, acidosis, and minimal pulmonary vascular resistance/systemic vascular resistance alterations.^18^ Benefits of general anesthesia include the avoidance of profound sympathetic block, commonly seen in spinal and epidural techniques, and the ability to concomitantly use transesophageal echocardiography.^19^ Regional (epidural and spinal) anesthesia preserves the mother's baseline ventilation and perfusion state. Epidural anesthesia also allows for continuous anesthetic and analgesic infusion, allowing for adjustment during delivery to minimize pain. Additionally, regional anesthesia can be reversed more expediently, while general anesthesia can take longer for recovery.

Because of the patient's RV dysfunction, the team decided to use epidural anesthesia. General anesthesia is often avoided in patients with RV dysfunction because all induction and inhalational agents, apart from etomidate, tend to reduce RV contractility, and mechanical ventilation increases pulmonary vascular resistance.^20^ Also, for patients undergoing a planned cesarean section, regional anesthesia is preferred to general anesthesia. For patients with ACHD, the regional block can be started initially with a spinal approach and gentle titration of epidural block to minimize hemodynamic instability.^20^ With obstruction of the RV outflow, reducing catecholamine release and subsequent stress on the RV is imperative.

## CONCLUSION

All pregnant patients with complex ACHD and a WHO pregnancy risk classification of II-III or higher should be closely managed by a multidisciplinary team at a single tertiary center to ensure the safest outcome for mother and child. Predelivery consultations should be arranged with cardiology, obstetrics, and anesthesia. A primary and contingency plan, in case of maternal or fetal deterioration, should be made far in advance of delivery. Mode of delivery and anesthesia decisions should be made on a case-by-case basis, taking into account specialist opinions, patient wishes, and the nature of the ACHD. Patients with severe pulmonic stenosis can be managed conservatively through pregnancy with strict follow-up and specialty care. Our patient had severe pulmonic stenosis with a mean gradient >75 mmHg but did not require intervention during pregnancy. Appropriate medical management can result in a successful delivery, allowing intervention for pulmonic stenosis to be completed at a later date without the complicating factors of pregnancy.

## Notes

This article meets the Accreditation Council for Graduate Medical Education and the American Board of Medical Specialties Maintenance of Certification competencies for Patient Care and Medical Knowledge.
